# Metabolic and skeletal homeostasis are maintained in full locus GPRC6A knockout mice

**DOI:** 10.1038/s41598-019-41921-8

**Published:** 2019-04-12

**Authors:** Christinna V. Jørgensen, Sylvia J. Gasparini, Jinwen Tu, Hong Zhou, Markus J. Seibel, Hans Bräuner-Osborne

**Affiliations:** 1 Department of Drug Design and Pharmacology, Faculty of Health and Medical Sciences, University of Copenhagen, Universitetsparken 2, DK-2100 Copenhagen, Denmark; 2 Bone Research Program, ANZAC Research Institute, University of Sydney, Sydney, Australia

## Abstract

The G protein-coupled receptor class C, group 6, subtype A (GPRC6A) is suggested to have a physiological function in glucose and bone metabolism, although the precise role lacks consensus due to varying findings in different knockout (KO) mouse models and inconsistent findings on the role of osteocalcin, a proposed GPRC6A agonist. We have further characterized a full locus GPRC6A KO model with respect to energy metabolism, including a long-term high-dose glucocorticoid metabolic challenge. Additionally, we analyzed the microarchitecture of tibiae from young, middle-aged and aged GPRC6A KO mice and wildtype (WT) littermates. Compared to WT, vehicle-treated KO mice presented with normal body composition, unaltered insulin sensitivity and basal serum insulin and glucose levels. Corticosterone (CS) treatment resulted in insulin resistance, abnormal fat accrual, loss of lean mass and suppression of serum osteocalcin levels in both genotypes. Interestingly, serum osteocalcin and skeletal osteocalcin mRNA levels were significantly lower in vehicle-treated GPRC6A KO mice compared to WT animals. However, WT and KO age groups did not differ in long bone mass and structure assessed by micro-computed tomography. We conclude that GPRC6A is not involved in glucose metabolism under normal physiological conditions, nor does it mediate glucocorticoid-induced dysmetabolism in mice. Moreover, GPRC6A does not appear to possess a direct, non-compensable role in long bone microarchitecture under standard conditions.

## Introduction

G protein-coupled receptors (GPCRs) mediate essential communication between the exterior and interior of cells. The GPCR class C, group 6, member A (GPRC6A) is activated by L-amino acids and divalent cations^1,2^. In addition, some studies suggest that GPRC6A is activated by the bone-derived peptide, osteocalcin^3,4^, although we and others have failed to confirm osteocalcin as a GPCR6A agonist^5,6^. GPRC6A is widely expressed in many tissues, which combined with the broad range of suggested endogenous ligands, has led to several hypotheses on its physiological functions, with a main focus on a potential role in the regulation of fuel metabolism^7^.

To date, three different global GPRC6A knockout (KO) models have been generated and currently these constitute the best available tools to investigate the physiological roles of GPRC6A. The group of Quarles reported a complex dysmetabolic phenotype of their GPRC6A exon II KO mouse model, which included changes in systemic fuel metabolism, osteopenia as well as reduced testicular function in young mice^8,9^. Using the same mouse model, the group later observed reduced L-arginine-induced insulin secretion in islets isolated from GPRC6A KO mice compared to WT animals^10^. In contrast, in another GPRC6A KO mouse model where instead exon VI is deleted, L-arginine potently induced insulin secretion in KO mice. Hence this study did not support a role of GPRC6A in the mechanism underlying L-arginine-induced insulin secretion^11^. Furthermore, young GPRC6A exon VI KO mice did not exhibit glucose intolerance, insulin resistance, increased body fat or osteopenia under normal physiological conditions^11,12^. Only when fed a high-fat diet did GPRC6A exon VI KO mice develop a dysmetabolic phenotype compared to their WT littermates^13^. In agreement with the latter observations, the group of Murphy recently reported that basal glucose levels as well as glucose tolerance were similar in WT and a GPRC6A KO model in which the full *gprc6a* gene had been deleted^14^. In addition, we have observed a slightly attenuated L-arginine-induced GLP-1 secretion in these KO mice compared to WT littermates^15^.

The current study aimed to expand the characterization of the full locus GPRC6A KO model with respect to energy metabolism and bone microarchitecture. We used supraphysiological doses of corticosterone (CS), the main glucocorticoid in rodents, as a metabolic challenge due to the pronounced effects of glucocorticoids on fuel metabolism, serum osteocalcin levels (the proposed GPRC6A ligand) and skeletal metabolism and structure^16–18^. As we observed a significant reduction in osteocalcin levels in the GPRC6A KO mice under basal conditions, we also determined whether the lack of GPRC6A is associated with a bone phenotype by determining the tibial microstructure of 9–12, 47–50 and 62–66-week-old full locus GPRC6A KO mice and WT littermates.

## Results

### Body composition

**Figure 1:**
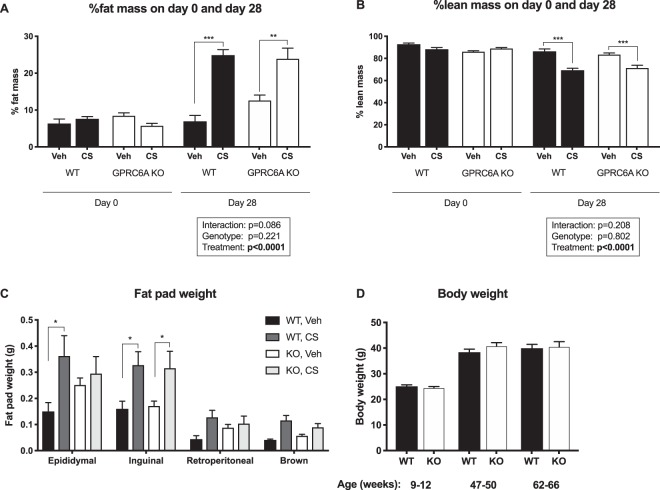
Body weight and composition of GPRC6A knockout (KO) mice and wildtype (WT) littermates. Total fat (**A**) and lean body mass (**B**) were measured by MRI in vehicle (veh)- and corticosterone (CS)-treated animals on day 0 and 28. There was no significant difference between the two vehicle-treated groups or between CS-treated WT and KO animals. (**C**) Changes in compartmental fat at endpoint demonstrated no significant differences between genotypes. (**D**) Body weight measured at the indicated ages. Data are presented as mean ± S.E.M. (n = 6–8). Data were analyzed by two-way ANOVA with Tukey’s *post hoc* test (**A–C**) or Student’s *t*-test (**D**). *P < 0.05, **P < 0.01, ***P < 0.001.

Body composition was similar in vehicle-treated full locus GPRC6A KO mice and WT littermates. This was the case both on day 0 and on day 28, corresponding to 8- and 12-week-old mice, respectively (Fig. 1A,B). Percent body fat mass increased proportionally in both vehicle-treated KO and WT animals over 4 weeks (WT: 7.0 ± 0.7% to 9.1 ± 1.3%, KO: 7.4 ± 0.7% to 10.6 ± 1.7%), while body lean mass changed only minimally over the same time period. GPRC6A KO and WT littermates did not display any statistically significant differences in body weight up to 66 weeks of age (Fig. 1D).

Mice treated with CS on the other hand became markedly obese within the 4-week treatment period (Fig. 1A). The fat mass increased by 3–4 fold from baseline. This was also reflected in the increased weight of the fat pads dissected at the end of the treatment (Fig. 1C). The CS-treatment also resulted in a rapid and significant loss of muscle mass (Fig. 1B). Of note, changes in fat and lean mass following CS-treatment were similar in both genotypes (Fig. 1A,B).

### Blood glucose and insulin levels, and insulin sensitivity

**Figure 2:**
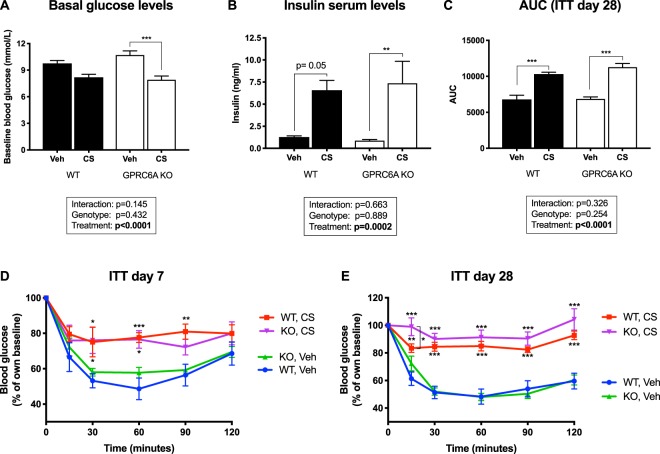
Glucose metabolism in GPRC6A knockout (KO) mice and wildtype (WT) littermates. (**A**) Basal blood glucose levels and (**B**) serum insulin levels at endpoint (day 28). Data are shown as mean ± S.E.M. (n = 6–8). (**D**) Insulin tolerance test (ITT) on day 7 and (**E**) day 28 of the treatment period. Data are presented as percentage of baseline blood glucose ± S.E.M. (n = 6–8). (**C**) The area under the curve (AUC) was calculated for each individual blood glucose response curve on day 28; data are shown as mean ± S.E.M for each group (n = 6–8). Two-way ANOVA with Tukey’s *post hoc* test. *P < 0.05, **P < 0.01, ***P < 0.001.

Basal blood glucose levels (WT: 9.8 ± 0.3 mmol/L; KO: 10.7 ± 0.5 mmol/L), serum insulin levels (WT: 1.3 ± 0.1 ng/ml; KO: 0.9 ± 0.1 ng/ml) and insulin sensitivity were similar in vehicle-treated GPRC6A KO and WT mice (Fig. 2).

With CS-treatment, both WT and GPRC6A KO mice developed insulin resistance as assessed by an insulin tolerance test (ITT) (Fig. 2D,E). This effect was seen as early as 7 days of CS-treatment and was similarly seen at the end of the treatment period (day 28). A small but statistically significant difference between genotypes was observed at the 15-minute time point on day 28, where the CS-treated WT mice displayed lower glucose levels than the CS-treated GPRC6A KO mice. No differences between the two genotypes were observed for the remaining time points resulting in a similar AUC in GPRC6A KO and WT mice (Fig. 2C). Compared to vehicle-treated mice, serum insulin levels were markedly increased in CS-treated animals at endpoint (WT: 6.6 ± 1.1 ng/ml; KO: 7.4 ± 2.5 ng/ml), with no difference between WT and KO mice. Basal blood glucose levels were decreased in CS-treated animals (WT: 8.2 ± 0.3 mmol/L; KO: 7.9 ± 0.4 mmol/L), again with no difference by genotype (Fig. 2A,B).

### Circulating osteocalcin levels and skeletal mRNA expression

**Figure 3:**
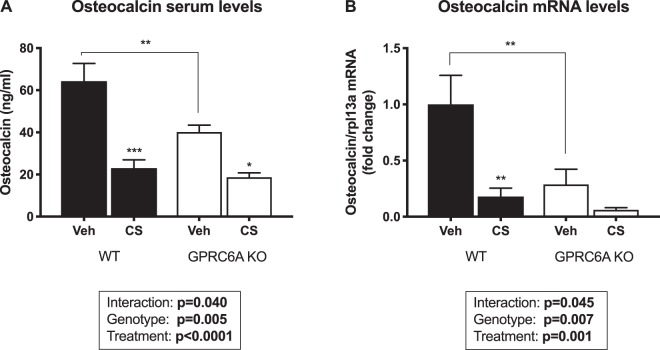
Vehicle (veh)-treated GPRC6A knockout (KO) mice exhibit significantly reduced osteocalcin levels. (**A**) Total osteocalcin serum levels measured on day 28. (**B**) Osteocalcin mRNA expression in the tibiae from vehicle- and corticosterone (CS)-treated GPRC6A KO mice and their WT littermates. Data are presented as mean ± S.E.M. (n = 6–8), Two-way ANOVA with Tukey’s *post hoc* test. *P < 0.05, **P < 0.01, ***P < 0.001.

Interestingly, total osteocalcin serum levels were significantly reduced in vehicle-treated KO mice compared to vehicle-treated WT mice (40.1 ± 3.3 ng/ml vs 64.4 ± 8.4 ng/ml, respectively) (Fig. 3A). To determine if the reduced amount of circulating osteocalcin could be due to a down-regulation of gene expression, we measured osteocalcin mRNA expression in the tibiae, which mirrored the circulating osteocalcin levels (Fig. 3B).

As expected, CS-treatment significantly reduced osteocalcin serum levels in both WT and GPRC6A KO genotypes (WT: 23.0 ± 4.0 ng/ml; KO: 18.7 ± 2.1 ng/ml) (Fig. 3A). Again, this was reflected in the osteocalcin mRNA levels (Fig. 3B).

### Trabecular and cortical microstructural parameters are unaffected by genotype

**Figure 4:**
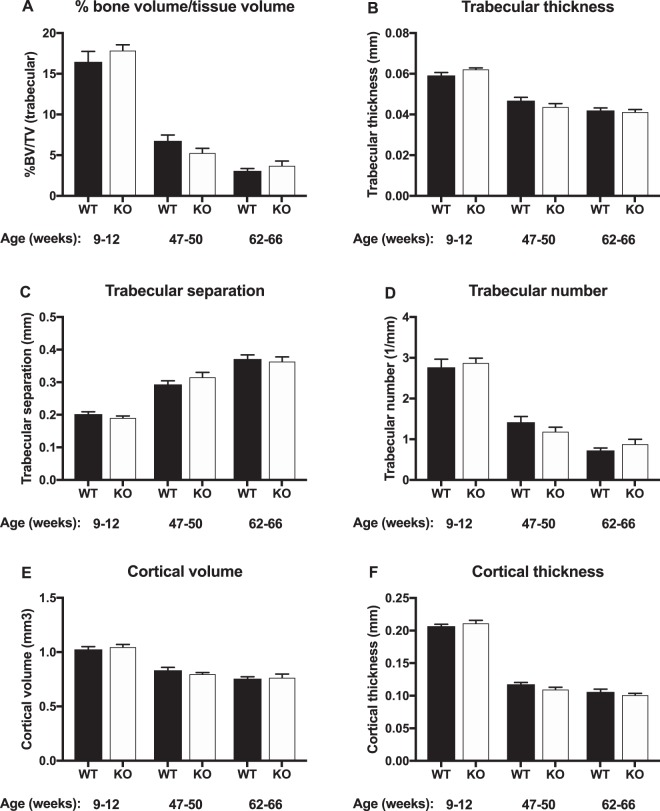
Trabecular and cortical microstructural parameters determined by micro-CT in full locus GPRC6A knockout (KO) mice and wildtype (WT) littermates. Data are presented as mean ± S.E.M. (n = 8–13), no statistical differences were observed between genotypes using Student’s *t*-test.

Osteocalcin is a marker of bone remodeling, and specifically of osteoblast activity and bone formation^19^. Based on the reduced levels of osteocalcin we hypothesized that the full locus GPRC6A KO model would present with a bone phenotype. However, as previous bone phenotyping in GPCR6A KO mice had only been performed in young animals^8,12^, we decided to analyze bone microstructure in younger (9–12 week-old), middle-aged (47–50 week-old) and old (62–66 week-old) mice, expecting that the skeletal phenotype, should it exist, would become more apparent with age. As expected, bone mass decreased with age in all mice and both genotypes (Fig. 4). In contrast to our expectations, no statistically significant differences were observed between WT and GPRC6A KO mice. Thus, within each age group, bone volume (BV/TV), trabecular number, trabecular separation and trabecular thickness were all similar in the two genotypes. Furthermore, there were no significant differences in cortical bone volume or thickness between genotypes (Fig. 4E,F). In light of this obvious lack of genotype-related differences, histomorphometric analyses were not performed.

## Discussion

Due to its widespread expression and the wide range of proposed ligands, the GPRC6A receptor has been termed a ‘master receptor^20^. At the same time it has been questioned whether this receptor has any function in humans at all, as the most common human receptor variant is not present on the cell surface when recombinantly expressed^6,21,22^. Studies in an exon II KO model point towards a physiological function of the GPRC6A receptor in fuel and skeletal metabolism^8,20^, while our group has found no or only subtle effects in an exon VI KO and now also in a full locus KO model^7,11,12,15^. While both exon knockouts should lead to a non-functional receptor, we here expand the characterization of a full locus KO model to avoid exon-specific phenotypes. In addition, we challenged the mice with high-dose glucocorticoids in order to override potential compensatory mechanisms and determine if the loss of GPRC6A activity increases susceptibility towards CS-induced dysmetabolism.

Overall, in this full locus KO mouse model we see no indication of GPRC6A being associated with changes in glucose metabolism. The KO mice presented with normal basal serum insulin and glucose levels, unchanged insulin sensitivity, and a body composition that was similar to their WT littermates. These findings are in line with previous reports on exon VI and full locus KO models^11,13–15^ but contradict the findings by the group of Quarles, who describe a metabolic phenotype for the exon II KO model^8^. Reduced insulin serum levels were however not present in their later study^4^. In summary, vehicle-treated full locus KO mice do not exhibit metabolic anomalies under standard physiological conditions.

Treatment with high-dose glucocorticoids induced pronounced obesity, insulin resistance, loss of muscle mass and reduced levels of circulating osteocalcin, similar to what has been described by others^16,23^. Again, there were no metabolic differences between CS-treated GPRC6A KO mice and their WT littermates. Our results thus clearly demonstrate that GPRC6A functionality is not required for the adverse metabolic effects of CS-treatment. The GPRC6A-deficient mice are not more susceptible to develop steroid-induced dysmetabolism than WT mice under the experimental conditions used in this study. The reduced fasting blood glucose levels observed in CS-treated, insulin resistant animals agrees with previously published results^24^. In the fasted state, increased insulin levels are known to suppress hepatic glucose production, indicating that hepatic insulin sensitivity is maintained in both WT and GPRC6A KO mice. The hyperinsulinaemia seen in these animals may not only reflect an attempt by the pancreatic β cells to compensate for insulin resistance but may also be a consequence of impaired insulin clearance by the liver, which has recently been shown in rodents^24^.

The GPRC6A KO mice displayed markedly reduced levels of circulating osteocalcin, most likely due to a down-regulation in gene expression. This is in accordance with reported abnormalities in the exon II KO model but contrasts findings described in the exon VI KO model^8,9,12^. Osteocalcin is a specific product of the bone forming cells, the osteoblasts. The reduction in serum osteocalcin levels may be due to impaired osteoblast function in GPRC6A deficient animals, as previously shown by the group of Quarles^9^. The reduction in osteocalcin expression therefore led us to examine the microarchitecture of the long bones (tibiae) in young, middle-aged and aged GPRC6A KO mice and WT littermates. This being the first study performed in older GPRC6A KO mice, we again did not observe any differences in long bone microstructure compared to WT littermates in any of the age groups. It therefore appears that loss of GPRC6A in itself is not sufficient to induce a bone phenotype under physiological conditions in any of the age groups characterized here. Thus, the reduction in circulating osteocalcin levels in the vehicle-treated GPRC6A KO mice are likely not sufficient to be of biological relevance with respect to bone microarchitecture. Clearly, CS-treatment resulted in more pronounced osteocalcin reductions than the lack of GPRC6A in itself.

However, there might be alternative explanations for the lack of a bone phenotype in our studies. Redundant genes are known to give the organism genetic robustness^25^. The calcium-sensing receptor (CaSR) is the most closely related receptor to GPRC6A when comparing protein sequence, ligand preferences, widespread tissue expression and genomic organization^2,22^. This combined with the role of CaSR in bone development^26^ makes CaSR a potential compensator gene that can eliminate the possible effects due to the loss of GPRC6A.

Another explanation for the lack of both a metabolic and a bone phenotype could be that some genes have too small an effect on phenotype and therefore only can be detected at the population level and not in a group of mice^25^. In support of this statement, the metabolic and bone phenotypes reported for the exon II GPRC6A KO mice are relatively modest^8,9^, while we have not observed any statistically significant differences with respect to metabolism and bone physiology under standard physiological conditions in the exon VI KO model^11,12^ and now for the full locus model with this study. Several hundred SNPs have recently been reported for the human *gprc6a* gene. Some of these SNPs cause a loss-of-function^27^, such as the Arg57STOP SNP. We have found a small percentage of the popoulation to be homozygous for this SNP^21^. This makes it possible to test the phenotypic effects of the GPRC6A receptor in human populations, which addresses human relevance and effect size of GPRC6A deficiency directly. Indeed, we did not observe any major metabolic differences in the few humans harboring homozygous Arg57STOP in GPRC6A compared to the control group^21^, suggesting that the GPRC6A receptor only plays a minor role in regulation of metabolism in both mice and humans.

In conclusion, the current study shows that GPRC6A KO mice do not suffer from metabolic disturbances and that GPRC6A-deficiency does not increase susceptibility towards developing dysmetabolism in response to high-dose glucocorticoid treatment. Furthermore, this is the first study to analyze bone microarchitecture in older mice lacking the GPRC6A receptor, demonstrating that lack of GPRC6A is not associated with an appreciable skeletal phenotype in long bones. These data therefore, do not support a direct role of GPRC6A in bone physiology despite the reduction in osteocalcin levels. More studies are needed to clarify the potential involvement of GPRC6A in regulation of osteocalcin.

## Materials and Methods

### Animals

The genetically modified GPRC6A KO mouse model with a deleted region completely covering the GPRC6A locus was obtained from the Knockout Mouse Project (KOMP) repository and backcrossed into a C57BL/6N genetic background^14,15,28^. GPRC6A heterozygous breeding produced GPRC6A KO mice and WT littermate controls. Mice were fed standard chow *ad libitum* and were housed under controlled temperature (22 °C ± 2 °C) and humidity (55% ± 10%) in a 12-hour light, 12-hour dark cycle rodent facility. All experimental work was conducted in accordance with institutional guidelines and approved by the Animal Experiments Inspectorate in Denmark.

### Administration of corticosterone

8-week old WT and GPRC6A KO mice were treated with CS for 4 weeks. CS (C2505, Sigma-Aldrich) was dissolved in absolute ethanol without additives (02860, Sigma-Aldrich) and then added to the drinking water to a final concentration of 50 μg/ml and 1% ethanol, following a previously described protocol^23^. Vehicle-treated animals received drinking water with 1% ethanol. Drinking water was refreshed weekly. Glucocorticoid-treated animals displayed 4–6 fold higher serum CS levels than vehicle-treated mice (WT: 264.5 ± 34.5 ng/ml vs. 61.9 ± 14.8 ng/ml; KO: 293.8 ± 54.5 ng/ml vs. 48.5 ± 9.7 ng/ml).

### Body composition

Measurements of body fat and lean mass were obtained by scanning the mice in an EchoMRI 4in1 scanner (Echo Medical Systems, Houston, TX, USA). Scans were performed at baseline (day 0) and at the end of the treatment period (day 28).

### Insulin tolerance tests

ITTs were performed on day 7 and day 28 during the treatment period. Mice were fasted 6 hours prior to the test. A baseline blood glucose reading was obtained prior to an intraperitoneal injection of insulin (0.75 U/kg body weight; Humalog, Eli Lilly). Blood glucose levels were subsequently measured at 15, 30, 60, 90 and 120 min post-injection, using an Accu-check glucometer (Roche). Data are presented as percentage of baseline blood glucose levels ± S.E.M.

### Blood sampling and hormone measurements

Blood samples were taken by cheek punctures at night (day 21) and at sacrifice by cardiac puncture on 3-h fasted mice (day 28) under anesthesia (Hypnorm/Dormicum: 2.5 mg/ml fluanisone, 0.079 mg/ml fentanyl citrate, and 1.25 mg/ml midazolam. Subcutaneous injection of 10 ml/kg bodyweight). Blood samples were allowed to clot at room temperature for 20 min followed by 10 min centrifugation. Serum was collected and stored at −80 °C until further analysis.

Serum CS levels were determined in the samples collected at night using an EIA kit (K014-H1, Abor assays, Ann Arbor, USA). The assay has intra- and inter-assay coefficients of variation of 6.3% and 7.5% respectively at a concentration of 2,500 pg/mL, and a limit of detection of 16.9 pg/mL. Serum total osteocalcin and insulin levels were measured in cardiac blood with an Osteocalcin ELISA assay (#60–1305, Immutopics, San Clemente, USA) and Ultrasensitive Mouse Insulin ELISA kit (#90080, Chrystal Chem, Inc, Downers Grove, IL, USA), respectively. The Mouse Osteocalcin ELISA Kit has intra- and inter-assay coefficients of variation of 3.7% and 6.1% respectively at a concentration of 25 ng/mL and a limit of detection of 0.4 ng/mL. The detection limit of the Ultrasensitive Mouse Insulin ELISA kit is 0.05 ng/ml and intra- and inter-assay coefficients of variation are reported to be ≤10.0% All assays were run according to the protocols provided by the manufacturers.

### RNA extraction and real-time qPCR

The total RNA was extracted from the tibiae using the RNeasy Plus Universal Mini Kit (73404, Qiagen) according to manufacturer’s protocol. The tibiae were homogenized in a bullet blender in ice-cold QiaZol and with frozen bullets (three 32 mm and two 42 mm bullets). The RNA content was quantified with a NanoDrop. cDNA was synthesized using 1 μg RNA and the High Capacity cDNA Reverse Transcription kit (4368814, Applied Biosystem, Life Technologies). qPCR was performed using the Agilent Mx3005P qPCR System (Agilent Technologies, Santa Clara, CA) in a 20 μl reaction mixture containing 10 μl SYBR Green qPCR Master Mix (QUNT95074-012), 5 μl cDNA sample, 15 pmol of forward and reverse gene-specific primer-pairs (TagC, Denmark), and DNase-free water to a total volume of 20 μl. The specific sequences of the primer sets were: osteocalcin F: GCTCTGTCTCTCTGACCTCACA, osteocalcin R: TAGATGCGTTTGTAGGCGG, rpl13a: F: GGAGGGGCAGGTTCTGGTAT and rpl13a R: TGTTGATGCCTTCACAGCGT. The thermal profile was: initial denaturation at 95 °C for 30 s followed by 40 temperature cycles of 95 °C for 5 s, 60 °C for 15 s, and 72 °C for 10 s. The default cycle threshold (CT) value was used. Fold changes in mRNA expression levels were calculated according to the comparative CT method and normalized using the level of rpl13a mRNA as described previously^29^.

### Micro-computed tomography

Following euthanasia, tibiae were dissected from male mice aged 9–12, 47–50 and 62–66 weeks and fixed for two days in 4% paraformaldehyde/PBS. Micro-computed tomography was performed using a SkyScan 1172 (Bruker, Kontich, Belgium) at 100 keV, 100 μA and 590 ms using a 0.5 mm filter. In total, approximately 940 projections were collected per sample at a resolution of 7.4 μm per pixel. GPU Accelerated NRecon software (SkyScan, Kontich, Belgium) and CTAn software (CTAn1.8, Skyscan) were used for three-dimensional reconstruction and analyses, respectively. Two 1-mm-long regions of bone were analyzed. Trabecular bone was analyzed 0.5 mm distal from the growth plate while cortical bone was analyzed 2.5 mm from the growth plate. The scanning and analysis were performed blinded.

### Statistical analyses

All calculations and graphical presentations were performed using GraphPad Prism 7 software (GraphPad Software Inc, La Jolla, USA). The effects of corticosterone and mouse genotype were analyzed by two-way analysis of variance (ANOVA) followed by Tukey’s multiple comparison *post hoc* test. For the comparison of two genotypes, unpaired two-tailed Student’s *t-*test was used. Data are presented as means ± S.E.M. and significance level is set at P < 0.05 for all analyses.
